# Practical application of the interim internal threshold of toxicological concern (iTTC): a case study based on clinical data

**DOI:** 10.1007/s00204-022-03371-6

**Published:** 2022-09-23

**Authors:** Abdulkarim Najjar, Corie A. Ellison, Sebastien Gregoire, Nicola J. Hewitt

**Affiliations:** 1 Beiersdorf AG, Unnastrasse 48, 20245 Hamburg, Germany; 2 The Procter & Gamble Company, 8700 Mason Montgomery Road, Cincinnati, OH 45040 USA; 3 L’Oreal Research & Innovation, 1, Avenue Eugène Schueller, 93601 Aulnay-sous-Bois, France; 4 Cosmetics Europe, Avenue Herrmann-Debroux 40, 1160 Brussels, Belgium

**Keywords:** iTTC, Cosmetics ingredients, Decision tree, Systemic toxicity, PBPK, Dermal exposure, UV filter, Risk assessment

## Abstract

We present a case study that provides a practical step-by-step example of how the internal Threshold of Toxicological Concern (iTTC) can be used as a tool to refine a TTC-based assessment for dermal exposures to consumer products. The case study uses a theoretical scenario where there are no systemic toxicity data for the case study chemicals (avobenzone, oxybenzone, octocrylene, homosalate, octisalate, octinoxate, and ecamsule). Human dermal pharmacokinetic data following single and repeat dermal exposure to products containing the case study chemicals were obtained from data published by the US FDA. The clinical studies utilized an application procedure that followed maximal use conditions (product applied as 2 mg/cm^2^ to 75% of the body surface area, 4 times a day). The case study chemicals were first reviewed to determine if they were in the applicability domain of the iTTC, and then, the human plasma concentrations were compared to an iTTC limit of 1 µM. When assessed under maximum usage, the external exposure of all chemicals exceeded the external dose TTC limits. By contrast, the internal exposure to all chemicals, except oxybenzone, was an order of magnitude lower than the 1 µM interim iTTC threshold. This work highlights the importance of understanding internal exposure relative to external dose and how the iTTC can be a valuable tool for assessing low-level internal exposures; additionally, the work demonstrates how to use an iTTC, and highlights considerations and refinement opportunities for the approach.

**Supplementary Information:**

The online version contains supplementary material available at 10.1007/s00204-022-03371-6.

## Introduction

The threshold of toxicological concern (TTC) is a human risk assessment tool for evaluating exposure to chemicals with limited toxicological data. When the TTC is used for a risk-based evaluation, the TTC value is compared to human external exposure estimates (e.g., mg/kg/day), because the TTC is based on the distributions of No Observed Adverse Effect Levels (NOAELs) derived from oral doses administered in preclinical toxicity studies (i.e., mg/kg/day). If human exposure to a chemical is below the relevant TTC value, it can be judged “with reasonable confidence, to present a low probability of a risk” (Munro et al. 1996). While the TTC has been a valuable tool for addressing low-level external exposures, it has been suggested that a TTC based on plasma concentration, referred to as “internal TTC” (iTTC) would be an improvement on the external dose TTC (Bessems et al. 2017; Blackburn et al. 2020; Ellison et al. 2019, 2020; Rogiers et al. 2020; SCCS 2021c). Given the same external exposure, differences in absorption, distribution, metabolism, and excretion (ADME) parameters, such as clearance and oral absorption, lead to differences in plasma concentrations. Therefore, comparing internal concentrations accounts for the ADME differences, providing a more accurate assessment of exposure.

The Cosmetics Europe Long Range Science Strategy program is leading a multi-stakeholder collaboration project that aims to develop robust iTTC values that can be used for the human safety assessment (Ellison et al. 2019, 2020). Physiologically based pharmacokinetic (PBPK) modeling is being used to convert the distribution of oral NOAELs from the TTC database to a distribution of internal exposures. Due to the complexity to derive iTTC values that are robust for risk assessment, an interim iTTC has been proposed as a single value, first tier, conservative internal exposure concentration (Blackburn et al. 2020). An interim iTTC of 1 μM was suggested by Blackburn et al. (2020) based on experiences from the pharmaceutical industry, an in-depth review of published non-drug chemical/receptor interactions, and an analysis of ToxCast™ data. Chemicals excluded from the interim iTTC approach comprise the original TTC exclusions (inorganic substances, proteins, nanomaterials, radioactive substances, organosilicon substances, metals, organometals, steroids, high potency carcinogens, and bioaccumulative compounds for example, poly-halogenated-dibenzodioxins, -dibenzofurans, and -biphenyls (EFSA et al. 2019; EFSA and WHO 2016; Worth et al. 2012; Yang et al. 2017), as well as chemicals expected to have potent chemical/receptor interactions with the estrogen and/or androgen receptor. Blackburn et al. (2020) also illustrated the application of the interim iTTC in the context of a toxicological structure–activity relationship (SAR) metabolism-based read-across assessment. In the Blackburn et al.’s example, toxicity data are available to support the safety of the metabolites, whereby estimates of systemic exposure to residual parent compound resulting from consumer product exposure are modeled and compared to the interim iTTC value (Blackburn et al. 2020). Ellison et al. (2019) have also provided a hypothetical example for the use of iTTC in a metabolism-based read across. Additionally, Ellison et al. (2019) explored a hypothetical example where the iTTC could be used to refine a TTC-based assessment for dermal exposure to a consumer product. In their example, a hypothetical chemical is used in a facial moisturizer and is non-mutagenic and non-genotoxic, but lacks systemic toxicity data. The hypothetical chemical has sufficient ADME data available to make predictions of internal exposure which would then be compared to an iTTC.

Here, we present a case study that examines the possible use of iTTC as a tool that can be used to refine a TTC-based assessment for dermal exposures to consumer products. We expand on this assessment by utilizing a set of case study chemicals which have robust human dermal pharmacokinetic data that were collected in well-designed clinical studies. The availability of such human PK data is limited for consumer product; thus, this dataset represents a unique opportunity to evaluate the utility of iTTC. Additionally, we walk through the practical application of iTTC in a step-by-step manner, and discuss inclusion and exclusion criteria, possible refinements, and other necessary considerations.

## Results and discussion

### Case study problem formulation and purpose

Human PK data following single and repeated dermal exposure to products containing oxybenzone, homosalate, octisalate, octinoxate, avobenzone, octocrylene, and/or ecamsule have recently become available (data further described below) (Matta et al. 2019, 2020). The case study uses a theoretical scenario of no systemic toxicity data for these chemicals (i.e., ignoring existing data for repeat dose toxicity, developmental toxicity, and reproductive toxicity). The current case study evaluates the possible use of the interim iTTC value of 1 μM to cover the theoretical limited data for systemic toxicity for the case study chemicals. The use of iTTC in this case study is aimed at developing and implementing non-animal-based tools for safety assessment. The intention is to demonstrate the possible use of iTTC as a tool that can be used to refine a TTC-based assessment for dermal exposure to a consumer product. Two constraints that will be applied to the case study are: (1) only PK data from the Matta et al. (2019, 2020) will be utilized; (2) no additional kinetic modeling (e.g., PBPK modeling) will be done.

### Case study chemicals

The seven case study chemicals are avobenzone (CAS No. 70356-09-1), oxybenzone (CAS No. 131-57-7), octocrylene (CAS No. 6197-30-4), homosalate (CAS No. 118-56-9), octisalate (CAS No. 118-60-5), octinoxate (CAS No. 5466-77-3), and ecamsule (CAS No. 92761-26-7). These are data-rich chemicals for which there is a large amount of existing mammalian toxicity data. Moreover, case study chemicals have previously been reviewed by global regulators and independent scientific advisory boards and served as the basis for establishing the guidelines for the safe use of these chemicals (CIR 2019; ECHA 2020; ECHA 2022; Nash 2006; SCCS 2020; SCCS 2021a; SCCS 2021b). However, we are using a theoretical scenario where there are no systemic toxicity data for these chemicals.

The case study chemicals were chosen, because they all have been tested in well-designed clinical studies and have a robust set of human PK data following dermal application to consumer products (further described below). The availability of these human PK data represents a unique opportunity to evaluate the utility of iTTC for a dataset, where external and internal exposure are carefully monitored. Additionally, there is an opportunity to directly compare the human PK data to the interim iTTC limit of 1 μM without any additionally modeling, such as PBPK modeling, which could introduce uncertainty into the evaluation. Given that this is the first case study that compares the [interim] iTTC to true human exposures, we wanted to minimize additional sources of uncertainty.

### Data source for human dermal PK data for case study chemicals

The systemic PK following dermal application of the 7 case study chemicals was evaluated by the US FDA in two separate clinical studies involving the use of commercially available sunscreen products (Matta et al. 2019, 2020). The first clinical study (Matta et al. 2019) evaluated four products that contained avobenzone, oxybenzone, octocrylene, and/or ecamsule in different concentrations, while the second clinical study (Matta et al. 2020) evaluated four products that contained homosalate, octisalate and/or octinoxate (note that one formulation was common between the two clinical studies). The clinical studies utilized a sunscreen application procedure that followed maximal use conditions consistent with current US sunscreen labeling and applied product as 2 mg/cm^2^ to 75% of the body surface area, four times a day for 4 days. This application procedure was followed every day in Matta et al. (2019) and on days 2–4 in Matta et al. (2020); product was only applied 1 time on day 1 in Matta et al. (2020). The concentration of the case study chemicals varied within the different formulas and is summarized in Table 1 along with the product form and overview of the clinical study design. The reported concentrations were in ng/ml; therefore, the values were converted to molarity concentrations using the molecular weight of the chemicals and considering the mean, and minimum and maximum observed data.

**Table 1:** Summary of exposure conditions for case study chemicals

Data source for exposure to case study chemicals	Case study chemical	Concentration of case study chemical in formula (%)	Name of formulation in original Matta et al.’s reference	Maximal use conditions exposure scenario	Average dermal exposure (mg/kg/application)	Average dermal exposure (mg/kg/day)^a^
Matta et al. (2019)	Avobenzone	3	Spray 1	2 mg/cm^2^ to 75% of body surface area, 4 times per day for 4 days	11.7	46.9
		3	Spray 2		11.8	47.4
		3	Lotion		11.6	46.2
		2	Cream		7.4	29.8
	Oxybenzone	6	Spray 1		23.5	93.8
		5	Spray 2		19.7	79.0
		4	Lotion		15.4	61.6
	Octocrylene	2.35	Spray 1		9.2	36.8
		10	Spray 2		39.5	158.0
		6	Lotion		23.1	92.4
		10	Cream		37.2	149.0
	Ecamsule	2	Cream		7.4	29.8
Matta et al. (2020)	Avobenzone	3	Lotion	2 mg/cm^2^ to 75% of body surface area at 0 h on day 1 and 4 times on day 2 through day 4 at 2 h intervals	11.3	45.1
		3	Aerosol spray		11.4	45.6
		3	Nonaerosol spray		11.5	45.8
		3	Pump spray		11.4	45.4
	Oxybenzone	4	Lotion		15.0	60.1
		6	Aerosol spray		22.8	91.1
	Octocrylene	6	Lotion		22.5	90.2
		10	Aerosol spray		38.0	151.9
		10	Nonaerosol spray		38.2	152.8
	Homosalate	15	Aerosol spray		56.9	227.8
		10	Nonaerosol spray		38.2	152.8
		10	Pump Spray		37.9	151.5
	Octisalate	5	Nonaerosol spray		19.1	76.4
		5	Aerosol spray		19.0	75.9
		5	Pump spray		18.9	75.7
	Octinoxate	7.5	Nonaerosol spray		28.7	114.6
		7.5	Pump Spray		28.4	113.6

^a^Exposure calculations based on dosing regimen of 4 applications per day

### Dermal exposure to case study chemicals following maximal use conditions

By considering product application rates, the concentration of a chemical in the formula, and study participant demographics (body surface area and body weight reported by Matta et al.), it is possible to calculate the dermal exposure to the case study chemicals. The external exposure (Table 1) for all the case study chemicals in all of the different product types is above the external TTC limits irrespective of Cramer Classification [Cramer Class I limit = 0.046 mg/kg BW/day; Cramer Class III limit = 0.0023 mg/kg BW/day (SCCS 2021c)]. As such, the ‘traditional’ TTC based on external exposure would be insufficient to cover the theoretical scenario where there are no systemic toxicity data for these chemicals. Conceptually, a TTC-based assessment for dermal exposure could be refined in the same manner as an assessment using chemical-specific oral data; specifically, the dermal exposure could be refined using information on dermal penetration relative to oral absorption. A refinement for a TTC-based assessment could be viewed as more difficult given that the oral TTC values are based on a population of chemicals within the Cramer Class, rather than a single chemical. That being said, even with chemical-specific assessments, oral absorption data are often not available. Several potential procedures for refining dermal exposure to account for dermal penetration for TTC-based assessments have been proposed, but none have been explicitly accepted by the Scientific Committee on Consumer Safety (SCCS). The interim iTTC (discussed below) is not subject to these same concerns, because the proposed value of 1 µM is based on human internal exposures rather than animal external doses.

### Interim iTTC inclusion/exclusion criteria and workflow

**Fig. 1:**
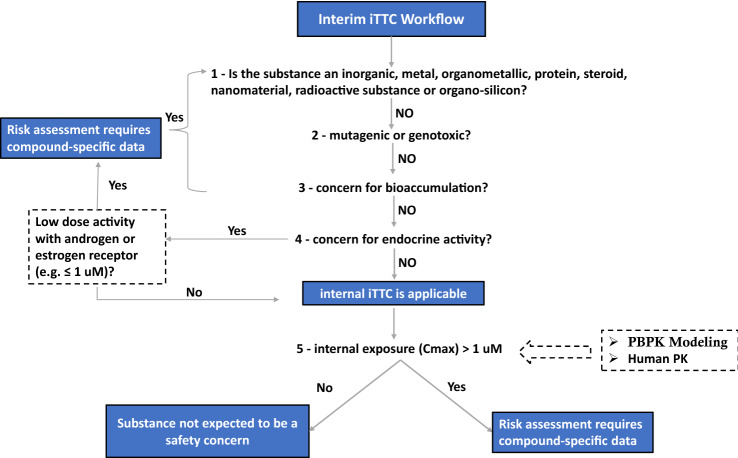
Interim iTTC workflow and inclusion/exclusion criteria

An outline of the interim iTTC inclusion and exclusion criteria and workflow are shown in Fig. 1. As discussed in detailed by Blackburn et al. (2020), exclusion from the interim iTTC approach include chemicals expected to have potent chemical/receptor interactions with the estrogen and/or androgen receptor in addition to the original exclusions (type of substance, mutagenicity/genotoxicity, and bioaccumulation) for the non-cancer TTC. Thus, prior to comparing an internal exposure to the interim iTTC value of 1 μM, it is necessary to make sure that a chemical is in the applicability domain of the interim iTTC. In the below section, we walk through each of these inclusion/exclusion criteria within the context of the case study.

### Criterion 1

Following EFSA and WHO guidance (EFSA and WHO 2016), substances that are inorganic, metals, organometallics, proteins, steroids, nanomaterials, radioactive substances, or organo-silicones should be excluded.

#### Result for case study chemicals

The case study chemicals are not any of these types of substances and are therefore not excluded based on this criterion.

### Criterion 2

As for the original non-cancer TTC approach, chemicals with potential for mutagenicity and genotoxicity are excluded from the iTTC approach.

#### Result for case study chemicals

Based on conclusions from the SCCS, reports from the Cosmetic Ingredient Review (CIR) and National Industrial Chemicals Notification and Assessment Scheme (NICNAS) and data summaries in the European Chemical Agency (ECHA) database, existing data from in vitro and in vivo studies indicate that the case study chemicals do not present a mutagenic or genotoxic hazard.

### Criterion 3

The EFSA and WHO guidance (EFSA and WHO 2016) exclude substances that are predicted to bioaccumulate in humans after direct exposure to the chemical. In the context of TTC, EFSA defines bioaccumulation as “the increasing retention of a chemical by an organism over time, in comparison with the concentration in the environmental media to which the organism is exposed (air, water, soil, food, etc.)” (EFSA 2012). It is further explained that chemicals that can bioaccumulate should be excluded from TTC. Specific examples that have been provided include TCDD and its structural analogues (poly-halogenated dibenzodioxins, dibenzofurans, and -biphenyls), where their half-lives in human are in the order of years. For example, the half-lives of tetra-, penta-, hexa-, hepta-, and octachlorodibenzo-*p*-dioxin in human ranged from 5.8 to 132 years (Flesch-Janys et al. 1996; Geyer et al. 2002; Golor et al. 1992; Poiger and Schlatter 1986; Ritter et al. 2011). More recently identified chemicals with high bioaccumulation potential, such as the perfluorinated chemicals, could also be excluded. EFSA states that bioaccumulation potential may be impacted by octanol–water partition coefficient, steric hindrance of metabolism, and stability of chemical bonds (EFSA 2012); however, no specific guidance is given on how to evaluate for bioaccumulation potential. Tonnelier et al. (2012) suggest the use of a PBPK model as a way to screen chemicals for human bioaccumulation potential. In cases when actual PK data are available, the PK data can be used to help answer the question of bioaccumulation, as illustrated by Ellison et al. (2020).

#### Result for case study chemicals

The case study chemicals are not anticipated to represent a concern for human bioaccumulation based on multiple lines of evidence which is explained in the following discussion. The case study chemicals do not resemble TCDD or its structural analogues and do not contain metabolic blocking groups (i.e., moieties that slow the rate metabolism, e.g., poly-halogenated). When using a toxicokinetic approach (Tonnelier et al. 2012) to estimate human bioaccumulation potential, by accounting for fraction unbound to plasma protein and hepatic metabolism, the case study chemicals have low human bioconcentration factors (see supplemental Table 1). Finally, human PK data from the US FDA clinical studies (Matta et al. 2019, 2020) demonstrate that the case study chemicals are readily excreted from the body, as indicated by terminal half-lives being on the order of days, and not months-to-years, which would be the case for chemicals that do bioaccumulate. Although we explore these multiple lines of evidence for case study purposes, it should be noted that in practice, this criterion for TTC has typically been addressed via a careful review of the structure to ensure that it does not resemble chemicals that are known to bioaccumulate (e.g., TCDD and it structural analogues). Finally, a main concern for bioaccumulating substances and the use of an iTTC value is the potential for a disconnect between plasma concentration and toxicity (for example, highly bioaccumulating chemicals partition into fat depots creating the potential for increasing bioburden with time that is disconnected from plasma concentration).

### Criterion 4

Blackburn et al. (2020) indicate that substances that raise concerns for endocrine activity (involving the estrogen and/or androgen receptors as targets) at low doses should be excluded from the interim iTTC based on the fact that estrogen and androgen receptors have agonists that can interact at concentrations below 1 μM. However, chemicals with activity relevant to in vivo effects at low concentrations were confined to a few well-characterized structural groups and many in vitro positives were not relevant to low-dose in vivo effects when evaluated against legacy in vivo data (Blackburn et al. 2020). Agonists can be steroid ring structures and non-steroid ring structures—both which can be identified via in silico alert approaches such as the DART decision tree (Wu et al. 2013). More specifically, the category 2 rules from Wu et al. (2013) can be invoked: 2a rules for steroid nucleus-derived estrogen receptor and androgen receptor binders, and 2b rules for non-steroid nucleus-derived estrogen receptor and androgen receptor binders, flavone- and mycoestrogen-related derivatives. Quantitative SAR (QSAR) models (Mansouri et al. 2016, 2018, 2020; Wedebye et al. 2016) can be used to predict potency toward the estrogen and androgen receptors and help identify chemicals which may be potent agonist and thus excluded from the interim iTTC. The US EPA CompTox Chemicals Dashboard (https://comptox.epa.gov/dashboard/) and Danish QSAR Database (https://qsar.food.dtu.dk/) contain QSAR models for the endocrine and androgen receptor. When in vitro bioactivity data (e.g., ToxCast^™^ data) are available for a chemical, they should be combined into predictive models as done in the Endocrine Disruptor Program (EDSP). These types of models are available in the US EPA CompTox Chemicals Dashboard (Browne et al. 2015; Kleinstreuer et al. 2017; Mansouri et al. 2016, 2020). The reason for this is that a single in vitro test (particularly those addressing only receptor binding) may significantly over-estimate in vivo potency. These concerns are clearly addressed in the discussion of the EDSP models. In cases when legacy in vivo animal data are available they can be used to answer the question of estrogen and androgen receptor activity. Alternatively, in vivo structure activity data across structurally related chemicals may be helpful when legacy in vivo data for the specific chemical are not available. When screening for this criterion, it is important to recall that chemical activity for a receptor occur across a range of potencies and the intent of this criterion is to identify and exclude chemicals that have potential activity toward the estrogen or androgen receptor in a concentration range near or below the interim iTTC (1 μM).

#### Result for case study chemicals

For the purpose of the case study, we will discuss the different tiers (i.e., in silico structural alerts, QSAR modeling, in vitro-based prediction modeling, and conclusions from in vivo data) that can be used to evaluate this criterion. In practice, it is generally agreed that in vitro data outweigh in silico predictions, and both are outweighed by in vivo data. Based on the structures of the case study chemicals, oxybenzone, homosalate, and octisalate would be identified as being a non-steroid nucleus-derived estrogen receptor or androgen receptor binder, flavone- and mycoestrogen-related derivative [Category 2b alert from the DART decision tree (Wu et al. 2013)]. Within the US EPA CompTox Dashboard, the QSAR prediction models, COMPARA (Consensus) and CERAPP Potency Level (Consensus) can inform on androgen and estrogen receptor activity for the case study chemicals. When utilizing these QSAR models for the case study chemicals, all the chemicals are predicted as inactive for androgen receptor activity and inactive (avobenzone, octinoxate, ecamsule) or very weak (oxybenzone, octocrylene, homosalate, octisalate) for estrogen receptor activity. The classification of very weak estrogen receptor activity corresponds to an activity concentration between 20 and 800 μM. Many of the case study chemicals have been tested in ToxCast^™^ assays, but only two chemicals (oxybenzone and homosalate) have been tested in a sufficient number of androgen and estrogen receptor assays to enable computational modeling of the data. As previously discussed (Browne et al. 2015; Kleinstreuer et al. 2017), estrogen and androgen-mediated responses are evaluated through a suite of in vitro bioactivity assays that measure different parts of the adverse outcome pathway, and it is necessary to integrate the results of these assays via computational modeling to accurately capture the activity of a chemical; reviewing the in vitro bioactivity data without a computational approach can lead to false interpretation of the bioactivity. The ToxCast Pathway Model in the CompTox Dashboard has been developed as a computational model to integrate the results of the in vitro bioactivity assays and provide a prediction for androgen and estrogen pathway activity. The model predicts an area under the curve (AUC) value for the potential of a chemical to show androgenic and estrogenic activity normalized with respect to a positive control chemical, estradiol. For androgen receptor activity, oxybenzone and homosalate are both predicted to be inactive by the ToxCast Pathway model and to have potential values of 0.0645 and 0.0217, which corresponds to an ‘inconclusive’ category. As described by the model developers, “very low bioactivity scores in the inconclusive range are not biologically relevant” (Browne et al. 2015). The endocrine activity potential of oxybenzone, octocrylene, and homosalate has been reviewed carefully by the (SCCS 2020) and they concluded that while there are indications of endocrine activity from some data, the data are either insufficient, inconclusive, or at best equivocal. As such, there is a lack of current evidence to regard these chemicals as endocrine disrupting substances or to derive a toxicological point of departure based on endocrine disrupting properties for use in human health risk assessment; in some cases, further investigations may be warranted. Importantly, in the context of the current case study, the preponderance of the evidence indicates that these chemicals are not expected to be potent agonists/antagonists for the estrogen receptor or androgen receptor and thus are in the applicability domain of the interim iTTC.

### Step 5: Determine internal exposure

If a chemical is determined to be in the applicability domain for the interim iTTC, the next step in the workflow is to determine the internal exposure in humans. As discussed in Ellison et al. (2019), there are multiple ways to determine internal exposure, including PK data, PBPK modeling approaches, ‘simple’ PK equations, and biomonitoring. The characterization of internal exposure will differ depending on the risk assessment scenario that the interim iTTC is being applied to. For example, if the interim iTTC is being applied in the context of a metabolism-based read-across assessment to cover the low concentration of parent chemical that may be present in the systemic circulation, the internal exposure assessment will focus on the parent chemical, since this is where there are no systemic toxicity data. If the interim iTTC is being used in a biomonitoring context, then a particular analyte (parent or metabolite) of interest may be the focus of the internal exposure assessment. If the interim iTTC is to be used to assess a systemic toxicity data gap for a chemical that has no toxicity data for the parent or metabolites, then it will be necessary to understand the systemic exposure for the parent and metabolite, so that the internal exposure to each, either individually or as an aggregate, can be compared to the interim iTTC. In the last case, the decision to evaluate internal exposure to the parent and metabolite individually or as an aggregate should be guided by an assessment which reviews whether metabolism of the parent chemical is likely to be activating, detoxifying or neutral, with regards to toxicity potential. When metabolism is activating or detoxifying, the internal exposures to the parent and metabolite(s) may need to be separately compared to the interim iTTC, since they are likely to have different toxicity potentials; however, when metabolism is neutral, an aggregate internal exposure should be compared to the interim iTTC. Assessing the impact of metabolism (activating, detoxifying, and neutral) has been reviewed previously and it is clear that this is a challenging question. It is outside the scope of the current paper to discuss all the different strategies and the reader is directed to the related publications (Bauman et al. 2009; Beames et al. 2020; Kakutani et al. 2019; Kalgutkar and Dalvie 2015; Kalgutkar et al. 2005; Stepan et al. 2011; Thompson et al. 2016). It is worth noting that approaches to address this question can range in the type of data that are generated, including in silico-based approaches (metabolism and/or structural alerts), in vitro metabolism (identification and/or kinetics), in vitro bioactivity assessments for parent and metabolite, and use of existing toxicity data for metabolites.

#### Result for case study chemicals

The current case study evaluates the possible use of the interim iTTC value of 1 μM to cover the theoretical scenario where these chemicals had no data for systemic toxicity. In this context, the interim iTTC is being used to assess a systemic toxicity data gap for the parent chemical and possibly the metabolite(s). Use of the interim iTTC for the metabolites will depend on if there are existing toxicity data and/or PK data or suitable toxicological analogues for the metabolites. All of the case study chemicals, except for ecamsule, could be metabolized by the skin and/or in the systemic circulation (Guesmi et al. 2020). As such, use of the interim iTTC would require consideration of the internal exposure to parent and metabolite, except for ecamsule, since it is not metabolized (according to internal unpublished data and the summary in DrugBank). The PK data from Matta et al. (2019, 2020) were limited to quantification of parent chemical in plasma and no data related to metabolites were reported. Since the problem statement for the current case study is limited to the PK data from Matta et al. (2019, 2020), there is a data gap for internal exposure of the metabolites, except for ecamsule. Additional ADME and PK data for the case study chemicals along with PBPK modeling approaches could be used to estimate internal exposure to the metabolites; however, as stated, this is outside the scope of the current case study. Thus, the remainder of the discussion in this section will focus on the internal exposure to the parent chemicals.

**Fig. 2:**
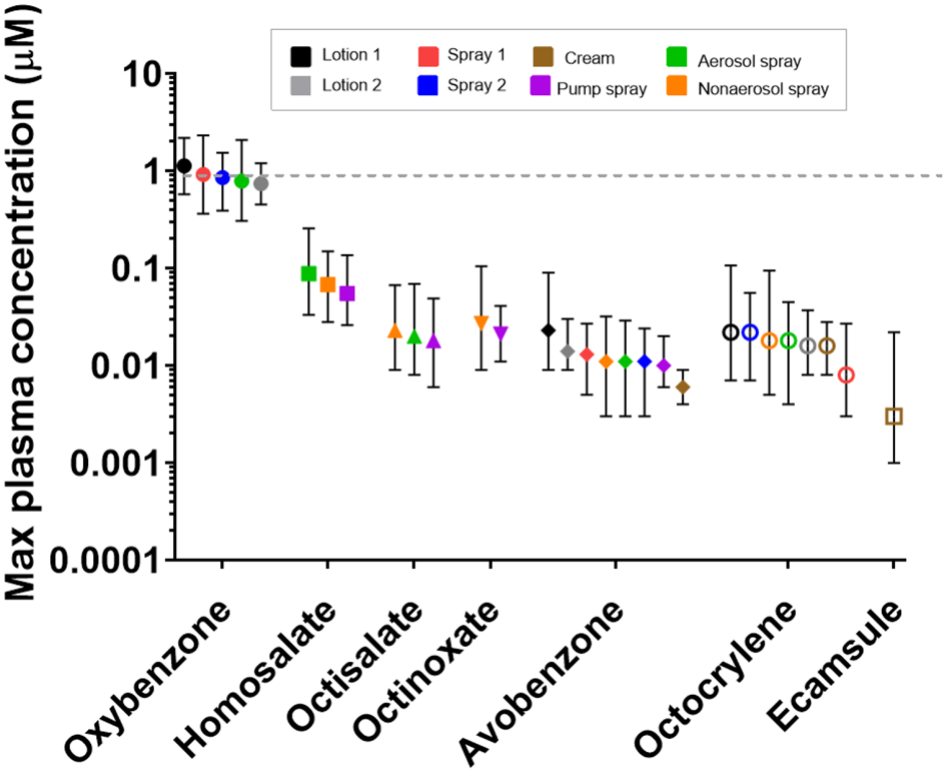
Maximum total (bound plus unbound) concentrations (*C*_max_) of case study chemicals in plasma following dermal application of different formulations under maximum exposure conditions. Symbols represent the average *C*_max_, while bars represent the lowest and highest observed *C*_max_ for an exposure scenario. The dotted lines denote the 1 μM interim iTTC concentration. Tabulated data are shown in Supplementary Table 2. All exposure data are from Matta et al. (2019, 2020)

Figure 2 summarizes the internal exposures measured in the clinical studies reported by Matta et al. (2019, 2020). The values are expressed as the average total (bound and unbound) *C*_max_ of the parent chemical in plasma following dermal application of different product formulations. The tabulated data from Fig. 2 are available in Supplementary Table 2. Figure 2 also includes the interim iTTC of 1 μM. The average total (bound and unbound) *C*_max_ for six of the case study chemicals (avobenzone, octocrylene, homosalate, octisalate, octinoxate, and ecamsule) was at least tenfold lower than the interim iTTC value of 1 μM and ranged from 0.003 μM (for ecamsule applied in a cream) to 0.088 μM (for homosalate applied in an aerosol spray). Moreover, the maximum total *C*_max_ measured for any of these six chemicals was 0.258 μM (for homosalate applied in an aerosol spray). There was one chemical, namely oxybenzone, for which the mean and/or maximum *C*_max_ exceeded the 1 μM interim iTTC threshold, regardless of which formulation it was applied in. The mean *C*_max_ values of oxybenzone ranged from 0.742 μM (lotion 2) to 1.131 μM (lotion 1) (which was the same composition as lotion 2, used in the second study). The highest maximum *C*_max_ value of oxybenzone was observed for Spray 1 (2.331 μM), although values for all six formulations exceeded the 1 μM interim iTTC value (1.203–2.331 μM).

### Consideration of uncertainty factors to apply to assessment

Blackburn et al. (2020) concluded that additional uncertainty factors do not need to be applied to the 1 μM interim iTTC value. However, they recommended to evaluate the need for uncertainty factors on a case-by-case basis, depending on the source of the exposure data and the structure of the substance. More specifically, when estimating the internal exposure of a chemical for comparison to the interim iTTC, it would be appropriate to consider either worst-case assumptions/estimates or the potential application of uncertainty factors. Another consideration is to evaluate whether the substance has a novel chemistry, since this would require additional conservatism and possibly more data to rule out the potential for potent biological activity.

#### Result for case study chemicals

For the current case study, additional uncertainty factors were considered unnecessary for several reasons. First, the internal exposure data were from well-designed clinical studies that had well-documented protocols, appropriate analytical methods, and transparent reporting of results. Second, the studies are designed to measure the exposure following maximal usage conditions consistent with current product labeling in the US (frequency—every 2 h; dose—2 mg/cm^2^ based on efficacy testing), and as such, it captures the highest end of possible exposure. An important consideration to evaluating the safety of a topically applied compound is the difference in skin penetration due to the formulation (Yang et al. 2020). In these studies, there were multiple formulation types compared (cream, lotion, spray, aerosols, etc.), which showed that the plasma concentrations did not vary markedly for cases when the chemical concentration was similar between the formulas or when the chemical concentration varied (Fig. 2). Therefore, there was a high confidence that the exposure measurements were representative of multiple exposure scenarios. A final area of conservatism in the overall approach is the fact that the internal exposures for the case study chemicals is a measure of total (bound and unbound) plasma concentration. When deriving the interim iTTC, Blackburn et al (2020) adjusted the in vitro bioactivity data to account for the bound vs unbound fraction in the assay, and as such, the interim iTTC of 1 μM M can be compared to the unbound fraction in the systemic circulation. However, Matta et al. (2019, 2020) only reported the total plasma concentration, thus making for a conservative assessment when the human PK data (bound plus unbound concentration) are compared to the interim iTTC (threshold for unbound concentration). A possible source of uncertainty relates to whether the plasma concentrations for the subjects reached steady state within the 4-day clinical study. The multiple areas of conservatism in the overall approach would likely account for the uncertainty related to steady-state.

### Comparison of exposure to the interim iTTC

The internal exposure to all except one case study chemical was an order of magnitude lower than the 1 μM interim iTTC threshold. This demonstrates that following maximal usage conditions consistent with current product labeling in the US, the exposure to the parent chemicals is sufficiently low. Oxybenzone was the only chemical that had internal exposures which exceeded the 1 μM interim iTTC threshold; however, the internal exposures to oxybenzone were very close to 1 μM. As discussed earlier in the case study, human PK data were unavailable for the metabolites; therefore, it is not possible to compare corresponding metabolite concentrations to the interim iTTC. Ecamsule is the one case study chemical where an assessment of metabolite exposure would not be needed, since it is not metabolized (according to internal unpublished data and the summary in DrugBank). A possible refinement to the overall approach used in the case study would be to understand the fraction unbound of chemical in plasma, since this represents the fraction that may exert biological activity. For perspective, the in vitro fraction unbound of oxybenzone in human plasma was assessed in the ToxCast program and determined to be 0.01 using rapid equilibrium dialysis assay (Wambaugh et al. 2019). This illustrates how the total plasma concentration (bound plus unbound) for the case study chemicals is a conservative over-estimate of unbound plasma concentration; however, this refinement is outside the scope of the current case study, since it requires additional data on the fraction unbound.

## Summary

The current case study evaluated the possible use of the interim iTTC value of 1 μM to cover a hypothetical situation where there are no systemic toxicity data for the case study chemicals. We provide a practical step-by-step assessment that reviews a workflow for the use of the interim iTTC. This is the first case study using actual human PK data that demonstrates how to use an interim iTTC and discusses the considerations and refinement opportunities for the approach. The workflow involves the same exclusion criteria as the non-cancer TTC in addition to exclusion of chemicals that are expected to have potent chemical/receptor interactions with the estrogen and/or androgen receptor. The external exposure for all the case study chemicals was above the external TTC limits, and as such, the ‘traditional’ TTC based on external exposure would be insufficient to cover the theoretical scenario where there are no systemic toxicity data for these chemicals. The internal exposure to all except one case study chemical (oxybenzone) was an order of magnitude lower than the 1 μM interim iTTC threshold. No PK data were available for the metabolites, so it was not possible to compare corresponding metabolite concentrations to the interim iTTC. Ecamsule is the one case study chemical where an assessment of metabolite exposure would not be needed, since it is not metabolized. The case study highlighted the benefits, challenges, and opportunities with using internal exposure (e.g., interim iTTC) for a safety assessment.

## Supplementary Information

Below is the link to the electronic supplementary material.

Supplementary file1 (XLSX 59 KB)

## Data Availability

All data generated or analyzed during this study are included in this published article (and its supplementary information files).
