# Correction to: Practical application of the interim internal threshold of toxicological concern (iTTC): a case study based on clinical data

**DOI:** 10.1007/s00204-022-03398-9

**Published:** 2022-11-27

**Authors:** Abdulkarim Najjar, Corie A. Ellison, Sebastien Gregoire, Nicola J. Hewitt

**Affiliations:** 1 Beiersdorf AG, Unnastrasse 48, 20245 Hamburg, Germany; 2 The Procter & Gamble Company, 8700 Mason Montgomery Road, Cincinnati, OH 45040 USA; 3 L’Oreal Research & Innovation, 1, Avenue Eugène Schueller, 93601 Aulnay-sous-Bois, France; 4 Cosmetics Europe, Avenue Herrmann-Debroux 40, 1160 Brussels, Belgium

## Correction to: Archives of Toxicology 10.1007/s00204-022-03371-6

The article Practical application of the interim internal threshold of toxicological concern (iTTC): a case study based on clinical data, written by Abdulkarim Najjar · Corie A. Ellison · Sebastien Gregoire · Nicola J. Hewitt, was originally published electronically on the publisher’s internet portal on 23rd, September 2022 without open access. With the author(s)’ decision to opt for Open Choice the copyright of the article changed on 30th September 2022 to © The Authors 2022 and the article is forthwith distributed under a Creative Commons Attribution 4.0 International License, which permits use, sharing, adaptation, distribution and reproduction in any medium or format, as long as you give appropriate credit to the original author(s) and the source, provide a link to the Creative Commons licence, and indicate if changes were made. The images or other third-party material in this article are included in the article’s Creative Commons licence, unless indicated otherwise in a credit line to the material. If material is not included in the article’s Creative Commons licence and your intended use is not permitted by statutory regulation or exceeds the permitted use, you will need to obtain permission directly from the copyright holder. To view a copy of this licence, visit http://creativecommons.org/licenses/by/4.0.

